# Clinical outcomes of circumferential endoscopic submucosal dissection in esophageal squamous cell carcinoma > 50 mm: Retrospective cohort study

**DOI:** 10.1055/a-2760-6112

**Published:** 2025-12-19

**Authors:** Takakazu Miyake, Hiroaki Takahashi, Satoshi Okahara, Ayumu Takizawa, Takashi Yokoyama, Junichi Kodaira, Keisuke Ishigami, Shinji Yoshii, Hiroshi Nakase

**Affiliations:** 1 92187 Division of Gastroenterology and Hepatology, Department of Internal Medicine, Sapporo Medical University, Graduate School of Medicine, Sapporo, Japan; 2 692752 Department of Gastroenterology, Keiyukai Daini Hospital, Sapporo, Japan

**Keywords:** Endoscopy Upper GI Tract, Endoscopic resection (ESD, EMRc, ...), Dilation, injection, stenting, Barrett's and adenocarcinoma

## Abstract

**Background and study aims:**

This study aimed to assess both short-term safety and long-term outcomes of whole circumferential endoscopic submucosal dissection (WC-ESD) for esophageal squamous cell carcinoma (ESCC) with tumor length > 50 mm, and to evaluate effectiveness of aggressive prophylactic dilation combined with steroid therapy in preventing esophageal stricture.

**Patients and methods:**

A retrospective review was conducted on 67 patients who underwent WC-ESD for superficial ESCC between 2009 and 2019 at a single Japanese center. Patients were categorized into > 50-mm and ≤ 50-mm groups. The primary endpoint was incidence of post-ESD stricture in the main analysis excluding surgery/chemoradiotherapy cases within 90 days; sensitivity analysis included all patients. Secondary endpoints were number of dilations, steroid use, and long-term survival outcomes (overall survival [OS], disease-specific survival [DSS], relapse-free survival [RFS]).

**Results:**

Stricture incidence was not significantly different (> 50 mm vs. ≤ 50 mm: 13% vs. 18%, *P* = 0.708; sensitivity: 21% vs. 15%, *P* = 0.538). Although dilations were more frequent in the > 50-mm group (96% vs. 83%), median sessions were similar (8 vs. 7.5). Prophylactic dilation plus steroids reduced refractory strictures (25%→7%, *P* = 0.03). For pT1a-EP/LPM cases, 5-year OS, DSS, and RFS were 100% in both groups. In pT1a-MM/pT1b cases, survival was comparable, although OS tended to be lower with > 50-mm lesions (68.9% vs. 100%, *P* = 0.07).

**Conclusions:**

WC-ESD for superficial ESCC with a tumor length > 50 mm did not increase adverse events and provided comparable long-term survival. These findings support feasibility beyond guideline limits and emphasize further refinement of stricture prevention.

## Introduction

Endoscopic submucosal dissection (ESD) is a well-established treatment for superficial esophageal squamous cell carcinoma (ESCC), enabling en-bloc resection and accurate pathological evaluation 1 2 3 . Current guidelines recommend ESD for cT1a-EP/LPM lesions up to 50 mm in length. Particularly for circumferential lesions, this size limit is emphasized due to the high risk of postoperative esophageal stricture (ES) 4 .

However, in clinical practice, whole circumferential ESD (WC-ESD) is frequently performed beyond these criteria. A recent Japanese multicenter survey reported that more than half the institutions had performed WC-ESD under extended indications, including lesions > 50 mm or with suspected submucosal invasion 5 . These real-world practices reflect a need for evidence regarding the feasibility and safety of WC-ESD beyond guideline limitations.

Notably, the current recommendation regarding the 50-mm threshold is largely based on limited evidence, such as the single-center study by Miwata et al., which included only 19 patients undergoing circumferential ESD 6 , and long-term outcomes after WC-ESD for larger lesions remain unclear.

Therefore, this study aimed to evaluate short-term safety and long-term outcomes of WC-ESD, focusing on lesions > 50 mm in length, and to assess risk of esophageal stricture (ES) and its management in a real-world cohort.

## Patients and methods

### Study design and population

This retrospective observational study was conducted in a single center at the Keiyukai Second Hospital, Hokkaido, Japan. We retrospectively analyzed 766 consecutive patients who underwent ESD for superficial cESCC (cT1N0M0) between January 2009 and June 2019. Exclusion criteria were described as follows: patients who had undergone prior chemoradiotherapy for ESCC, those with multiple ESCC lesions treated in a single ESD session, or patients who had two or more ESD procedures for ESCC during the study period.

To evaluate clinical outcomes of WC-ESD with a longitudinal extension > 50 mm, patients were classified into two groups: WC-ESD with a longitudinal extension > 50 mm (> 50 mm group) and ≤ 50 mm (≤ 50 mm group). Each group was further categorized into pT1a-EP/LPM and pT1a-MM/pT1b (SM1/SM2) subgroups based on post-ESD histopathological findings for prognostic analysis. Primary analysis excluded patients who initiated surgery or chemoradiotherapy (CRT) within 90 days after ESD when estimating short‑term stricture incidence. In the sensitivity analysis, all patients were included, counting events that occurred before surgery/CRT initiation and censoring at that time. Long-term outcome analysis (overall survival [OS]/disease-specific survival [DSS]/recurrence-free survival [RFS]) included all patients, with surgery/CRT considered part of real-world management. In addition, descriptive data from non-WC-ESD cases were included as supplementary information to aid interpretation of patient backgrounds ( **Supplementary Table 1** ).

Information about the study was posted on the hospital website, allowing participants to opt out. Patients who did not opt out of the study were considered to have provided tacit consent to participate. The study protocol was reviewed and approved by the Review Board of Keiyukai Second Hospital (approval date: May 8, 2021). The study adhered to the ethical principles outlined in the Declaration of Helsinki 7 .

### Indication for ESD

Extent of the lesions was determined using image-enhanced endoscopy and Lugol’s chromoendoscopy. Invasion depth of ESCC was estimated using white-light non-magnifying endoscopy to assess changes on the lesion surface. Narrow band imaging magnifying endoscopy was used to further evaluate abnormal microvessels according to the Japan Esophageal Society classification.

Computed tomography (CT) and endoscopic ultrasound (EUS) were used to rule out lymph node metastasis, and positron emission tomography/CT was performed as necessary. As recommended by the guidelines, we performed ESD for clinically diagnosed T1a-EP/LPM 4 .

### ESD procedures

Sedation was achieved using midazolam (5–10 mg), pethidine (0–50 mg), and dexmedetomidine hydrochloride (loading dose of 6 μg/kg over 10 minutes, maintenance at 0.2–0.7 μg/kg/h, with adjustments based on patient condition. Electrosurgical units used were ICC-200 (Intelligent Cut and Coagulation; Erbe, Tübingen, Germany) and VIO3 (Erbe). The circumference of the lesion was first marked using spotty electrocautery with a needle knife 1 mm from the tumor border and confirmed by Lugol-unstained regions. Normal saline mixed with glycerin (ranging from 10 to 100 mL, with an average of 40 mL) and epinephrine (0.005%) was injected into the lesion. Glycerin concentration was 10% of the weight/volume (Chugai Pharmaceutical Co., Ltd., Tokyo, Japan). The marked area was incised along its perimeter using a hook knife (Olympus, Tokyo, Japan). Submucosal dissection of the lesion was performed after mucosal incision. Two expert endoscopists (HT and SO) performed all ESD procedures. In circumferential ESD cases, the clip-with-line method 8 and endoscopic submucosal tunnel dissection 9 were utilized when appropriate.

### Definition of WC-ESD

**Fig. 1:**
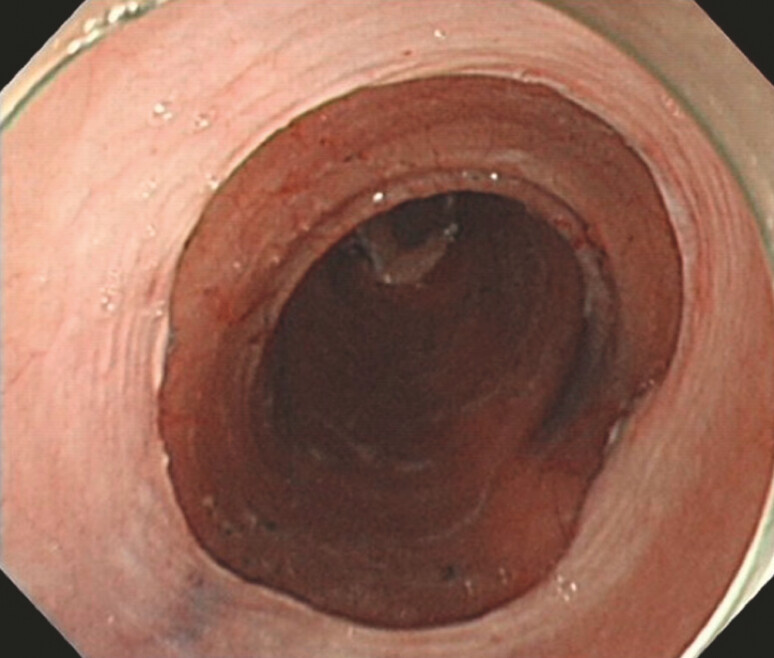
A complete circumferential esophageal mucosal defect following ESD.

WC-ESD was defined as a complete circumferential esophageal mucosal defect following ESD ( Fig. 1 ). Patients with remaining esophageal mucosa were classified as non-WC-ESD 10 .

### Steroid treatment

To prevent ES after ESD, patients with mucosal defects involving more than three-quarters of the esophageal circumference were immediately injected endoscopically with triamcinolone acetonide (TA) (Kenacort-A, 40 mg/mL, Bristol-Myers Squibb, Anagni, Italy) ( **Supplementary Fig. 1** ). TA was diluted with 0.9% NaCl to a final concentration of 10 mg/mL. Aliquots (0.5 mL) were injected at the base of the artificial ulcer using a 25G, 3-mm needle (TOP Corporation, Tokyo, Japan). Injections were initiated at the distal edge of the ulcer base and evenly spaced at 10-mm intervals toward the proximal edge, taking care to avoid injury to the muscularis propria 11 . Oral prednisolone was started at 30  mg/day on the second day post-ESD and continued with a gradually tapering prednisolone dose (30, 25, 20, 15, 10, and 5 mg/day). Systemic steroid administration was discontinued after 12 weeks. At the time when we started WC-ESD at our hospital, even in cases of WC-ESD, there were some cases in which we did not perform steroid injections and oral steroids. Furthermore, we adopted oral steroids at our hospital in 2018. Regarding local steroid injections, we perform them on the ulcer base on the day of WC-ESD, but not during subsequent dilation.

### ES

ES was defined as inability to pass a standard diagnostic gastroscope (which had a diameter of 9.8 to 10.2 mm; GIF-H260, GIF-H290Z, or GIF-HQ290; Olympus Medical Systems, Tokyo, Japan) through the stenotic segment. In addition to endoscopic findings, clinical symptoms such as dysphagia were also taken into account when diagnosing ES, if documented in the medical records.

### Dilation therapy

Dilation therapy was administered to the patients with dysphagia resulting from ES. Prophylactic dilation was also performed in patients who underwent WC-ESD ( **Supplementary Fig. 1** ). The interval between dilations ranged from 1 to 4 weeks. Dilation was repeated until dysphagia symptoms resolved and a gastroscope (8.9–9.9 mm) could pass without resistance 4 weeks after the final session. Dilation therapy was conducted in the outpatient department using a silicone bougie (Maloney, Medovations, Milwaukee, Wisconsin, United States) or a balloon dilator (CRE Fixed Wire 15 mm/16.5 mm/18 mm, Boston Scientific, Massachusetts, United States). We defined refractory strictures as “cases that underwent dilatation six times or more.”

### Pathological examination

Resected specimens were sectioned perpendicularly at 2-mm intervals and examined by a board-certified gastrointestinal pathologist. Tumor size, histological type, differentiation, lymphovascular invasion, and lateral and deep margins were evaluated according to the 11th Edition of the Japanese Classification of Esophageal Cancer 12 . Complete resection (R0) was defined as en-bloc resection with pathological tumor-free lateral and vertical margins. Histopathological analysis confirmed that curative resection revealed pT1a-EP/LPM with no lymphovascular invasion.

### Follow-up protocol and additional treatment

After ESD, patients with pT1a-EP/LPM ESCC underwent regular endoscopic surveillance. The first follow-up endoscopy was conducted 6 months after ESD to assess mucosal healing, with subsequent endoscopic surveillance every 6 months. For patients with pT1a-MM and T1b ESCC, with or without additional treatment, follow-up included measurement of tumor markers, endoscopy and CT every 3 to 6 months for 5 years.

We recommend additional treatment with surgery or CRT (50.4 Gy external-beam irradiation with 5-fluorouracil and cisplatin) for patients with pT1b ESCC, positive lymphovascular invasion, or positive/unclear VM (VM1/VMX). Some patients were monitored without additional treatment based on their refusal or overall condition.

### Outcome

The primary endpoint was incidence of short-term adverse events (AEs), particularly ES, in the > 50-mm and ≤ 50-mm groups. This was assessed in the primary analysis, which excluded patients who initiated surgery or CRT within 90 days after ESD. A sensitivity analysis was also performed, including all patients and counting events that occurred before surgery/CRT initiation. Additional short-term outcomes included number of dilatations and use of steroid therapy.

Secondary endpoints focused on long-term outcomes, including OS, DSS, and RFS in the > 50-mm and ≤ 50-mm groups. Both groups were further stratified into the pT1a-EP/LPM and pT1a-MM/pT1b(SM1/SM2) subgroups.

OS was defined as time from the date of ESD to date of death from any cause other than esophageal cancer. DSS was defined as time from ESD to death, specifically from date of primary ESCC. Recurrence sites were categorized as local lymph nodes or distant organs.

### Statistical analysis

All statistical analyses were performed using SPSS Statistics version 22 (IBM Japan, Tokyo, Japan). For the primary analysis of short-term outcomes, patients who underwent surgery or CRT within 90 days after ESD were excluded when estimating incidence of post-ESD stricture. In sensitivity analyses, all patients were included, counting events occurring before surgery/CRT and censoring at the date of surgery/CRT initiation. For long-term outcomes, all patients were included, and surgery/CRT was regarded as part of real-world management.

Categorical variables were compared using the chi-square test or Fisher’s exact test, and continuous variables using the Mann-Whitney U test. Kaplan-Meier curves with log-rank tests were used for time-to-event analyses. Multivariate logistic regression was performed to identify factors associated with stricture and refractory stricture, adjusting for clinically relevant covariates. A two-sided *P* < 0.05 was considered statistically significant.

## Results

### Baseline characteristics

**Fig. 2:**
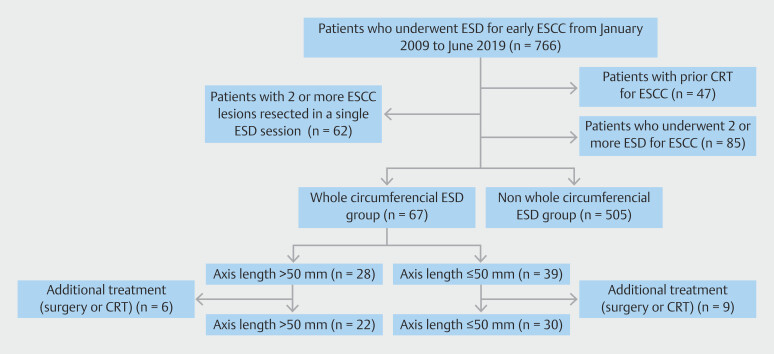
Patient flow chart of the study.

In total, 766 patients who underwent ESD for ESCC were included in this study. The exclusion criteria included 47 patients who had received prior CRT, 62 patients with multiple ESCC lesions resected in one ESD session, and 85 patients with two or more ESD treatments for ESCC. After applying these criteria, 67 patients were categorized into the WC-ESD group and 505 into the non-WC-ESD group ( Fig. 2 ). In the WC-ESD group, which was divided into two groups based on longitudinal extension longer than 50 mm or less, the > 50-mm group consisted of 28 patients and the ≤ 50-mm group consisted of 39 patients. Among the cases that underwent additional treatment with surgery or CRT, there were six cases in the > 50-mm group and nine cases in the ≤ 50-mm group. For short-term outcomes, including ES, the analysis was be conducted in the two groups (> 50-mm group: 28/≤ 50-mm group: 39) excluding cases that underwent additional treatment (surgery or CRT). Regarding long-term outcomes, we evaluated the two groups (> 50-mm group: 28/≤ 50-mm group: 39) including cases that underwent additional treatment (surgery or CRT). Median follow-up period was 61 months (interquartile range [IQR], 18–135), and was significantly shorter in the > 50-mm group than in the ≤ 50-mm group (64 [18–56] vs. 85 [26–146] months, *P* = 0.0244). The follow-up rate at the last observation was 85% at the 5-year follow-up.

Clinicopathological characteristics of patients in the > 50-mm and ≤ 50-mm groups are summarized in Table 1 . There was no significant difference in age between the two groups. The lesions were predominantly located in the middle thoracic esophagus in both groups, with 0-IIc being the most common macroscopic type.

**Table:** **Table 1** Baseline patient and lesion characteristics and pathological outcomes of WC-ESD (< 50-mm group and > 50-mm group).

	**Total (%)** **(n = 67)**	**Axis length > 50 mm (n = 28)**	**Axis length ≤ 50 mm (n = 39)**	***P* value **
Age at endoscopic treatment, median (IQR), years	71 (48–89)	73.5 (48–84)	70 (54–89)	0.494 ^†^
Sex				0.217 ^§*^
Male (%)	55 (82)	21 (75)	34 (87)	
Female (%)	12 (18)	7 (25)	5 (13)	
Tumor location				0.958 ^§*^
Ce (%)	3 (5)	1 (4)	2 (5)	
Ut (%)	12 (18)	5 (18)	7 (18)	
Mt (%)	36 (53)	16 (57)	20 (51)	
Lt (%)	16 (24)	6 (21)	10 (26)	
Macroscopic type				0.590 ^§*^
0-IIa	1 (2)	0 (0)	1 (3)	
0-IIb	8 (12)	2 (7)	6 (15)	
0-IIc	37 (55)	17 (61)	20 (51)	
Mixed	21 (31)	9 (32)	12 (31)	
Size of tumor, median (IQR), mm	65 (21–111)	71 (57–111)	57 (26–92)	0.0002 ^†*^
Depth of invasion				
pT1a (%)	46 (69)	20 (71)	26 (67)	0.679 ^§*^
pT1b (%)	21 (31)	8 (29)	13 (33)	
pT1a (%)				0.517 ^§*^
EP/LPM	28 (42)	11 (39)	17 (44)	
MM	18 (27)	9 (32)	9 (23)	
pT1b (%)				
SM1	6 (9)	1 (4)	5 (13)	
SM2	15 (22)	7 (25)	8 (20)	
Lymphatic invasion (%)	10 (15)	4 (14)	6 (15)	1.000 ^‡^
Venous invasion (%)	5 (8)	1 (4)	4 (10)	0.391 ^‡^
WC-ESD, whole circumferential endoscopic submucosal dissection. ^*^ Significant at *P* < 0.05. ^†^ Mann-Whitney's U-test. ^‡^ Chi-square test. ^§^ Fisher's exact test.				

### Long-term outcomes

**Fig. 3:**
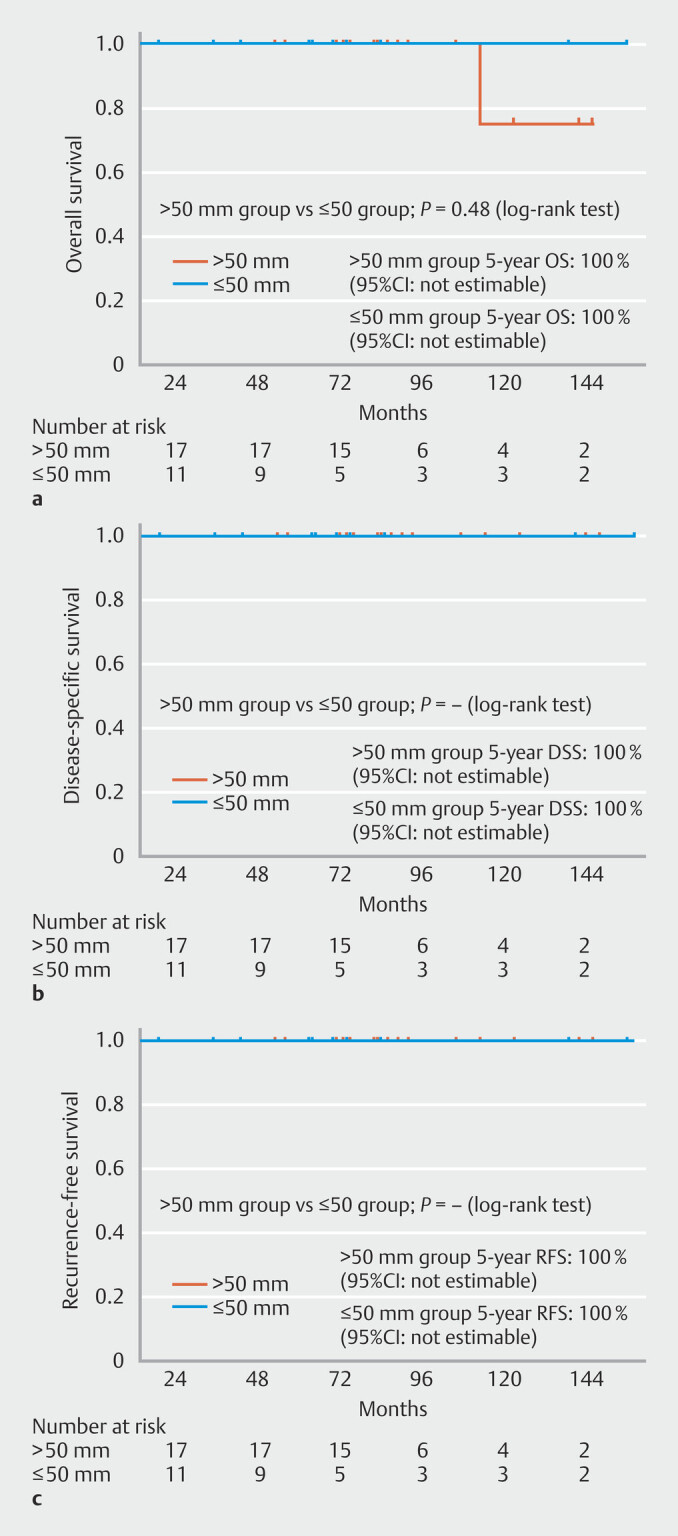
**a** Kaplan-Meier curves of OS for patients with pT1a-EP/LPM disease in the > 50-mm and ≤ 50-mm groups. **b** Kaplan-Meier curves of DSS for patients with pT1a-EP/LPM disease in the > 50-mm and ≤ 50-mm groups. **c** Kaplan-Meier curves of RFS for patients with pT1a-EP/LPM disease in the > 50-mm and ≤ 50-mm groups.

**Fig. 4:**
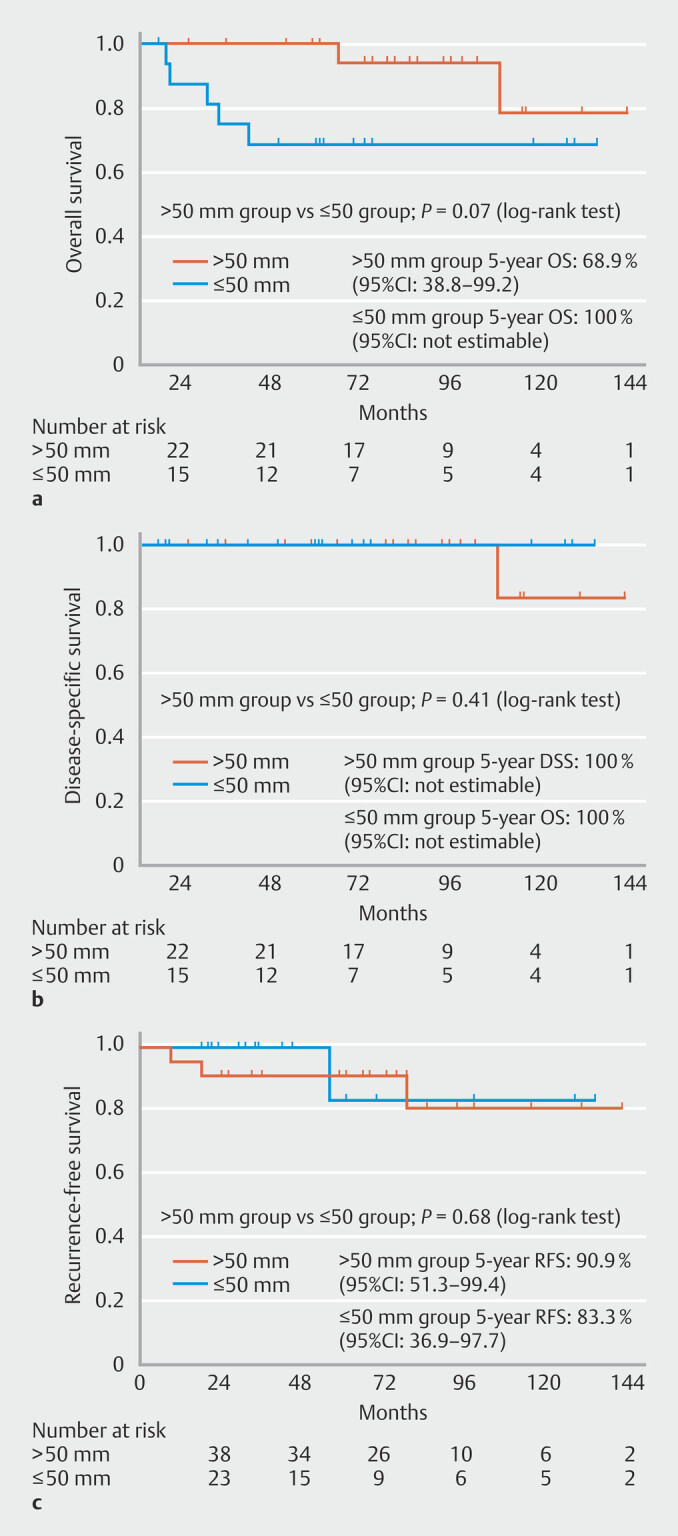
**a** Kaplan-Meier curves of OS for patients with pT1a-MM and pT1b disease in the > 50-mm and ≤ 50-mm groups. **b** Kaplan-Meier curves of DSS for patients with pT1a-MM and pT1b disease in the > 50-mm and ≤ 50-mm groups. **c** Kaplan-Meier curves of RFS for patients with pT1a-MM and pT1b disease in the > 50-mm and ≤ 50-mm groups.

For pT1a-EP/LPM cases (> 50-mm group 39%; 11/28, ≤ 50-mm group 44%; 17/39), 5-year OS was 100% (95% CI, not estimable), DSS was 100% (95% CI, not estimable), and RFS was 100% (95% CI, NE) in both groups ( Fig. 3 **a-c** ). In pT1a-MM and pT1b (SM1/SM2) cases (> 50-mm group 61%; 17/28), ≤ 50-mm group 56%; 22/39), 5-year OS was lower in the > 50-mm group than in the ≤ 50-mm group 68.9% (95% CI 38.8–99.2) vs 100% (95% CI-NE), respectively ( Fig. 4 **a** ), and there were no significant differences in 5-year DSS (100% vs 100%; 95% CI- NE and 95% CI-NE), respectively, and 5-year RFS between the two groups (90.9% and 83.3% and 95% CI 51.3- 99.4 and 95% CI 36.9- 97.7, respectively) ( Fig. 4 **b,c** ). The proportions of patients who required additional treatment in the > 50-mm and ≤ 50-mm groups were 21% (6/28) and 26% (10/39), respectively ( *P* = 0.777).

**Fig. 5:**
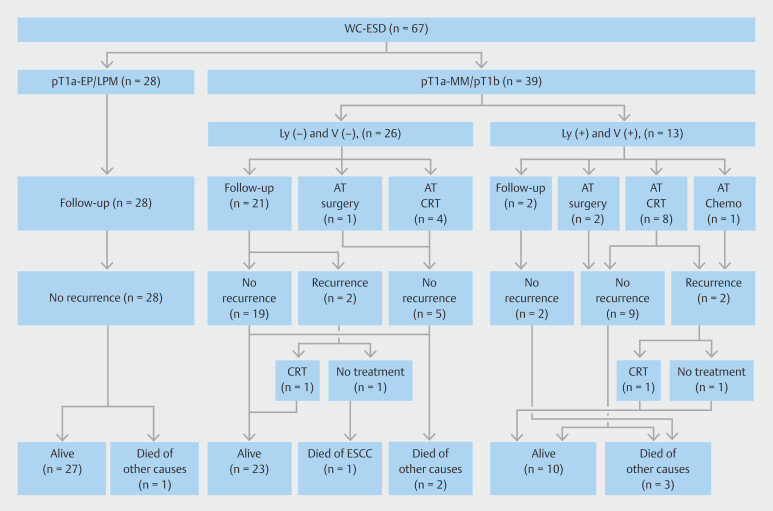
Clinical progression of WC-ESD with a longitudinal extension of > 50-mm cases. AT, additional treatment; Chemo, Chemotherapy.

The clinical course of the 67 patients who underwent WC-ESD is shown in Fig. 5 . No procedure-related deaths occurred in the WC-ESD group. However, one patient (2%) died of primary ESCC and seven died of unrelated causes.

Four patients in the WC-ESD group (6%, 4/67) experienced nodal recurrence; three had pT1b, and one had pT1a-MM with vascular invasion. Among them, two underwent CRT and one patient who declined additional treatment died of ESCC-related causes ( **Supplementary Table 2** ).

### Procedure characteristics and adverse events

Procedure characteristics and AEs in the > 50-mm and ≤ 50-mm groups are detailed in Table 2 . En-bloc resection was achieved in 100% of the cases in both groups. R0 resection rates were 95% in the > 50-mm group and 87% in the ≤ 50-mm group, respectively. The curative resection rate was not significantly different between the > 50-mm group and the ≤ 50-mm group (86% vs. 77%; *P* = 0.488).

Frequency of AEs was not significantly different between the > 50-mm group (18%, 4/22 patients) and the ≤ 50-mm group (13%, 4/13 patients; *P* = 0.708). These included ES. No cases of delayed bleeding and intraoperative perforation were observed in either group.

Results of ES treatment are presented in Table 2 . The proportion of patients in the > 50-mm group who required dilations was higher than in the ≤ 50-mm group, with no significant difference (96% vs. 83%, *P* = 0.226). Median number of dilations was not significantly different between the > 50-mm group and the ≤ 50-mm group (8 vs. 7.5) (IQR 0–39 vs. 0–53, respectively; *P* = 0.205). One patient in the > 50-mm group developed a perforation during dilation and required surgery. In the sensitivity analysis including patients who underwent surgery or CRT, stricture occurred in six of 28 patients (21%) in the > 50-mm group and six of 39 patients (15%) in the ≤ 50-mm group (OR 1.50; 95% CI 0.43–5.25; *P* = 0.538), consistent with the primary analysis. Local injection of steroids for preventing post-ESD strictures for prophylaxis was administered to 96% of the > 50-mm group and 87% of the ≤ 50-mm group. Two patients who received oral steroids were in the > 50-mm group patient (9% vs. 0%, *P* = 0.174).

**Table:** **Table 2** Procedure characteristics and adverse events of WC-ESD and outcomes related to treatment for stricture.

	**Total (%) (n = 52)**	**Axis length > 50 mm (n = 22)**	**Axis length ≤ 50 mm (n = 30)**	***P* value **
En-bloc resection (%)	52 (100)	22 (100)	30 (100)	-
R0 resection (%)	47 (90)	21 (96)	26 (87)	0.381 ^§^
Curative resection (%)	42 (81)	19 (86)	23 (77)	0.488 ^§^
Adverse events, n (%)	8 (15)	4 (13)	4 (18)	0.708 ^§^
Delayed bleeding, n (%)	0 (0)	0 (0)	0 (0)	-
Esophageal stricture, n (%)	8 (15)	4 (13)	4 (18)	0.708 ^§^
Intraoperative perforation, n (%)	0 (0)	0 (0)	0 (0)	-
Dilation, n (%)	46 (89)	21 (96)	25 (83)	0.226 ^§*^
Number of dilations, median (IQR), times	8 (0–53)	8 (0–39)	7.5 (0–53)	0.205 ^§*^
Complications after dilation (perforation), n (%)	1 (2)	1 (5)	0 (0)	0.423 ^§^
Refractory stricture, n(%)	35 (67)	16 (73)	19 (63)	0.473 ^§*^
Steroid, n (%)	47 (90)	21 (96)	26 (87)	0.381 ^§*^
Injection, n (%)	47 (90)	21 (96)	26 (87)	0.381 ^§*^
Oral, n (%)	2 (4)	2 (9)	0 (0)	0.174 ^§*^
IQR, interquartile range. ^*^ Significant at *P* < 0.05. ^†^ Mann-Whitney's U-test. ^‡^ chi-square test. ^§^ Fisher's exact test.				

## Discussion

This retrospective study evaluated clinical safety and long-term outcomes of WC-ESD for ESCC with a long axis length > 50 mm compared with WC-ESD for lesions ≤ 50 mm. No significant differences were observed between the two groups in the incidence of AEs, including ES, or in 5-year OS, DSS, and RFS. The proportion of patients requiring endoscopic dilation was numerically higher in the > 50-mm group than in the ≤ 50-mm group (93% vs. 77%, *P* = 0.0825), whereas median number of dilations was comparable between the groups. These results provide realistic evidence demonstrating the feasibility of WC-ESD beyond the limitations of current guidelines.

Current Japanese guidelines recommend WC-ESD for circumferential ESCC measuring ≤ 50 mm in length, primarily due to the high risk of postoperative strictures 6 . Nevertheless, multicenter surveys in Japan have shown that WC-ESD is often performed for larger lesions when technically feasible 5 . Most previous studies have focused on short-term safety in guideline-compliant cases 13 14 and long-term prognosis of patients who had received WC-ESD 15 . Furthermore, the guidelines provide no detailed recommendations for WC-ESD in other ESCC presentations, such as circumferential lesions exceeding 50 mm or those with submucosal invasion 4 . Therefore, our study addresses this evidence gap by providing real-world data on both short- and long-term outcomes of WC-ESD for lesions > 50 mm and demonstrating that preventive strategies that may mitigate associated risks. Data from the non-WC-ESD group were presented as supplementary information to aid interpretation.

Five-year OS for pT1a-MM and pT1b appeared numerically lower in the > 50-mm group than in the ≤ 50-mm group (68.9% vs. 100%, *P* = 0.07) and all deaths in these subgroups were from non-esophageal cancer causes. Median follow-up duration was 61 months (range, 8–148 months), with an 85.0% follow-up rate at 5 years. This follow-up rate reflects the challenges inherent in retrospective, real-world studies, in which the decreasing number of patients in long-term observation is common, but survival analysis appropriately accounted for censoring, and there was no significant difference in baseline characteristics between patients lost to follow-up and those retained, supporting the validity of our survival outcomes.

Curative resection rates in this study (68% in the > 50-mm group and 59% in the ≤ 50-mm group) were lower than expected. This may be due to the difficulty in accurately predicting depth of invasion in circumferential or large lesions, leading to underestimation of submucosal invasion even in lesions ≤ 50 mm. Some lesions initially judged as mucosal or muscularis mucosae were found to have submucosal invasion on histological analysis, leading to non-curative resection. This diagnostic limitation is consistent with previous observations in large, flat esophageal lesions 16 and underscores the need for improved preoperative staging techniques, such as high-frequency EUS or advanced imaging algorithms.

We found no significant differences in AEs, including ES, between the > 50-mm and ≤ 50-mm groups (13% vs. 18%). In a sensitivity analysis including patients who underwent surgery or CRT, stricture incidence remained similar (21% vs. 15%). The lower stricture rate compared with previous reports is likely attributable to prophylactic balloon dilation and steroid therapy, which reduced incidence of refractory strictures from 25% to 7%. These findings suggest that WC-ESD for lesions > 50 mm can be performed safely when such preventive measures are applied, although further improvements in stricture prevention remain necessary 17 18 19 .

This study has several limitations. First, its retrospective single-center design and relatively small sample size may limit generalizability of the findings. Second, the procedures were performed by only two endoscopists, which might have influenced treatment outcomes. Third, the imbalance in the number of cases between the > 50-mm and ≤ 50-mm groups complicates direct comparisons. Lastly, the balloon dilation data included both preventive and therapeutic procedures, making it difficult to precisely estimate the number of dilations required for stricture treatment. Large-scale registries or pooled analyses of real-world data could provide more robust evidence regarding safety and efficacy of WC-ESD for extensive esophageal lesions.

## Conclusions

In this large single-center cohort, WC-ESD for superficial ESCC in pT1a-EP/LPM case with tumor length > 50 mm showed no significant increase in AEs, including esophageal stricture, and the number of endoscopic dilations required was not substantially different from that for lesions < 50 mm when prophylactic steroid therapy and preventive dilation were implemented. In addition, long-term OS, DSS, and RFS were not substantially different between the two groups. These findings provide real-world evidence supporting the feasibility of WC-ESD beyond current guideline limits, while underscoring the need for ongoing strategies to reduce the high stricture rate.

## Supplementary Material

Supplementary Material Zusatzmaterial
