# Supplementary figures and images for: Clinical outcomes of circumferential endoscopic submucosal dissection in esophageal squamous cell carcinoma > 50 mm: Retrospective cohort study
Source: Endosc Int Open. 2025 Dec 19;13:a27606112. doi: 10.1055/a-2760-6112 (PMC12818186; doi:10.1055/a-2760-6112)

**Supplementary Fig. 1** Patient flow chart of steroid treatment and dilation.

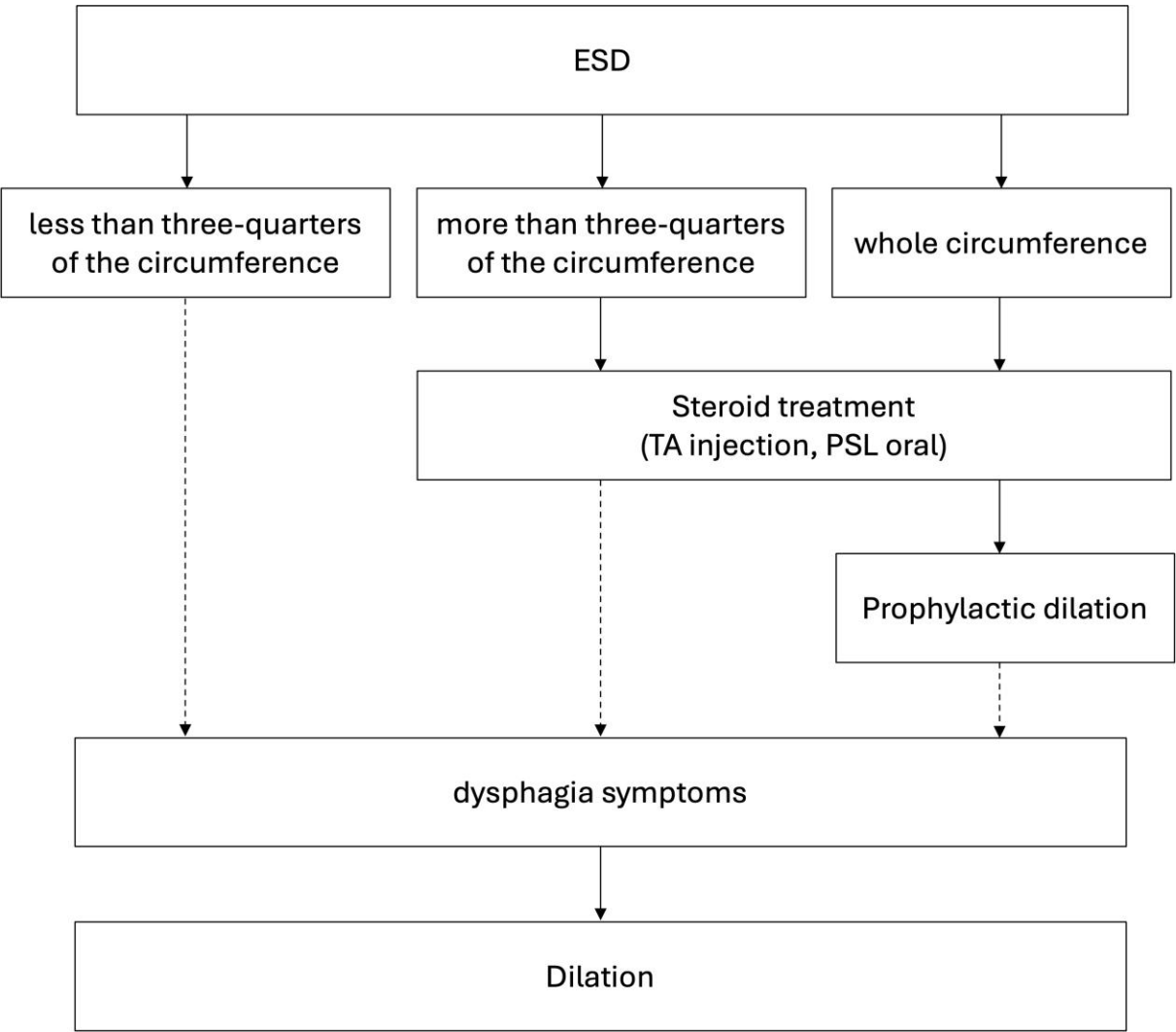

Supplement: Supplementary file 1 — Supplementary Material [file 10-1055-a-2760-6112_27654398.pdf]
